# A High-Efficiency TiO_2_/ZnO Nano-Film with Surface Oxygen Vacancies for Dye Degradation

**DOI:** 10.3390/ma14123299

**Published:** 2021-06-15

**Authors:** Huizhong Ma, Baofei Hao, Wentao Song, Jinpeng Guo, Mingyuan Li, Lan Zhang

**Affiliations:** School of Mechanics and Safety Engineering, Zhengzhou University, Zhengzhou 450001, China; haobaofei123@outlook.com (B.H.); Songwentao1234@outlook.com (W.S.); g98_1@outlook.com (J.G.); lmy97_221@outlook.com (M.L.)

**Keywords:** photocatalysis, ZnO/TiO_2_, heterojunction thin films, oxygen vacancies

## Abstract

Photocatalytic degradation of organic pollutants in water is a highly efficient and green approach. However, the low quantum efficiency is an intractable obstacle to lower the photocatalytic efficiency of photocatalysts. Herein, the TiO_2_/ZnO heterojunction thin films combined with surface oxygen vacancies (OVs) were prepared through magnetron sputtering, which was designed to drive rapid bulk and surface separation of charge carriers. The morphology and structural and compositional properties of films were investigated via different techniques such as SEM, XRD, Raman, AFM, and XPS. It has been found that by controlling the O_2_/Ar ratio, the surface morphology, thickness, chemical composition, and crystal structure can be regulated, ultimately enhancing the photocatalytic performance of the TiO_2_/ZnO heterostructures. In addition, the heterojunction thin film showed improved photocatalytic properties compared with the other nano-films when the outer TiO_2_ layer was prepared at an O_2_/Ar ratio of 10:35. It degraded 88.0% of Rhodamine B (RhB) in 90 min and 90.8% of RhB in 120 min. This was attributed to the heterojunction interface and surface OVs, which accelerated the separation of electron–hole (e–h) pairs.

## 1. Introduction

Advanced oxidation processes act as fiercely competitive technologies to remove recalcitrant organic pollutants based on chemical oxidation and have been widely recognized as efficient treatments for chemical contaminants, viruses, and bacteria in wastewater [1]. Heterogeneous photocatalysis is a main advanced oxidation process consisting of oxidative reductive reactions induced by the generation of highly reactive free radicals on the surface of photocatalysts in presence of light with energy equal or higher than photocatalytic materials’ forbidden bandgap [2]. Among various photocatalytic materials, titanium dioxide (TiO_2_) is a highly effective photocatalytic semiconductor owing to its prominent photocatalytic performance, superior chemical and thermal stability, low cost, and non-toxicity [3].

Anatase and rutile are two common polymorphic forms of TiO_2_. The photocatalytic efficiency of rutile is low due to the existence of numerous unordered grain boundaries, which act as the recombination centers of e–h pairs. The anatase phase of a TiO_2_ crystal has better photocatalytic properties owing to the higher electron lifetime, which is 100 times higher than that in the rutile phase [4]. The bandgap of TiO_2_ is wide (3.2 eV for anatase, 3.0 eV for rutile), which results in poor utilization ability of visible light [5,6]. Indeed, the limited charge transfer and high electron–hole recombination are the main factors hindering the photocatalytic performance of TiO_2_ [7]. To accelerate electron transport and reduce charge recombination of TiO_2_, many ways have been designed and employed, such as doping with transition metal or non-metal [8], increasing surface area [9], and constructing a proper heterostructure [10], etc. Among these different methods, constructing a proper heterostructure is one of the most effective strategies to improve the photocatalytic ability of TiO_2_ by accelerating the transfer of photo-generated e–h pairs at the interface between various semiconductor materials [11,12,13]. Zinc oxide (ZnO), as an excellent n-type semiconductor material, has been demonstrated as a preferred material to couple with TiO_2_ due to its facile synthesis, high photocatalytic efficiency, high abundance, and the fact that energy band position with ZnO is marginally more negative than that in TiO_2_ [14]. Besides, the slow surface separation of photo-generated e–h pairs is another obstacle that compromises the photocatalytic reaction efficiency [15,16]. Oxygen vacancies (OVs) act as adsorption, electron-trapping, and active sites for photocatalysis, which can accelerate the charge carrier separation on the surface by optimizing the surface properties [17].

Although TiO_2_ and ZnO are often fabricated as powders and exhibit superior catalytic properties, they must be removed from the treated water stream after the photocatalytic degradation to avoid harm and threat to human and aquatic lives. Moreover, the recovery of photocatalysts from the bulk of the water stream is costly, which is hard to achieve in largescale operations [18,19]. Many techniques have been developed to facilitate the catalyst recovery through immobilizing the catalyst on the substrate, such as chemical vapor deposition, sputtering, sol-gel, electrochemical deposition, atomic layer deposition, etc. Among them, magnetron sputtering is one of the most effective methods and can produce catalytically active thin films with superior structural and chemical properties because it offers several advantages including a high degree of order, uniformity of thickness and composition, good adhesion, and an easiness in composition control [20].

Many factors affect the structure and properties of photocatalysis films synthesized through magnetron sputtering including the working pressure, the O_2_/Ar ratio, the distance between substrate and target, the material type and temperature of the substrate, the sputtering power, and the target construction [21,22]. For example, Yu et al. synthesized the Ag-Ag_2_O-TiO_2_ photocatalyst thin films by the different magnetron sputtering methods and found that the RhB degradation ability of the TiO_2_ layer in heterostructured film prepared by DC magnetron sputtering and air oxidation was comparable to that prepared by reactive magnetron sputtering [23]. However, Yuan et al. showed that the degradation rate constant of the TiO_2_ film prepared by natural oxidation increased by four times compared with the TiO_2_ film prepared by reactive magnetron sputtering [24]. Moreover, ceramic TiO_2_ was also used as the target by some groups [5,25]. Thus, the effect of the O_2_/Ar ratio and target type on the photocatalytic performance of films needs further study.

In this work, TiO_2_ was deposited on the ZnO film via magnetron sputtering by using a metallic Ti target at different O_2_/Ar ratios for the first time, for comparison, a ceramic TiO_2_ was also used at the same Ar flow rate. The impact of the O_2_/Ar ratio and target type on the photocatalytic properties of TiO_2_/ZnO nano-films was investigated. As a result, ZnO coupled with TiO_2_ deposited at an O_2_/Ar ratio of 10:35 had the superior photocatalytic performance, which was due to the rapid transport of e–h pairs in the bulk and their fast separation on the surface.

## 2. Materials and Methods

TiO_2_/ZnO nano-films were deposited on the ITO glass substrate (3 cm × 3 cm) through magnetron sputtering (JGP-450, Shenyang ZKY Technology Development Co., Shenyang, China). Before deposition, the glass substrates were ultrasonicated in deionized water and ethanol for 20 min. The base pressure of the chamber was 6.0 × 10^−4^ Pa. The distance between the target and the substrate was about 15 cm. The target was pre-sputtered for 5 min to remove dirt and contaminants on the surface. ZnO film was first prepared by radio frequency magnetron sputtering of a ZnO target (99.999% purity) at 100 W of target power. The sputtering time was 60 min while sputtering pressure was fixed at 1.0 Pa. The flow rate of Ar gas was set at 35 sccm. Subsequently, TiO_2_ layer was deposited on ZnO layer through direct current magnetron sputtering of a metallic Ti target (99.999% purity) for 60 min at different O_2_/Ar gas ratios. The sputtering power was set to 100 W and the sputtering pressure was 1.0 pa. The values of O_2_/Ar gas flow rate were set at 0/35, 5/35, 10/35, and 20/35, namely, ZT1, ZT2, ZT3, and ZT4, respectively. To be compared, pure TiO_2_ layer was deposited on ZnO layer using a ceramic TiO_2_ target (99.999% purity) under sputtering power of 100 W, sputtering time of 60 min, sputtering pressure of 1.0 Pa, and O_2_/Ar gas flow rate of 0/35, namely, ZT5. All the ZnO/TiO_2_ nano-films were post-annealed in a muffle furnace at 450 °C for 2 h under ambient conditions.

The growth morphology and film thickness have been checked by scanning electron microscopy (SEM, Zeiss Auriga, Oberkochen, Germany). The crystalline phase of nano-films was studied by X-ray diffraction (XRD, Empyrean, Almelo, The Netherlands) in film mode with Cu-Ka radiation (1.5406 Å). The atomic force microscopy (AFM, SPM-9700, Kyoto, Japan) was used to obtain the surface roughness in tapping mode. The composition of these samples was determined by X-ray photoelectron spectroscopy (XPS, AXIS Spura, Manchester, UK) with Al-Ka (1486.6 eV) radiation operating at 120 W (voltage 12 KV, current 10 mA). Raman spectra were performed by laser Raman spectrometer (Raman, LabRAM HR Evo, Paris, France). The optical properties of the prepared TiO_2_/ZnO nano-films were measured by UV–vis absorption spectroscopy (UV-3150, Shanghai, China). The photocurrent transients of all the ZnO/TiO_2_ nano-films were measured in a three-electrode system. The TiO_2_/ZnO nano-films (1 cm × 1 cm) worked as the working electrodes. The Ag/AgCl and Pt foil were used as the reference electrode and work electrode, respectively. The current signal under the on/off mercury lamp (300 W) was recorded by electrochemical workstation (CHI660, Chenhua, Shanghai, China) in Na_2_SO_4_ solution (0.5 M in 50 mL). The photocatalytic properties of all the prepared five thin films were analyzed using a spectroscopic optical method. A total of 50 mL RhB solution with the concentration of 5 mg/L was used as the organic pigment. Samples (1 cm × 1 cm) were put into the solution at room temperature. First, the reaction solution was stirred before irradiation for 30 min in the absence of light. Then, the photocatalytic activity of the as-prepared samples was tested under the irradiation with a 300 W mercury lamp for 60 min, and the absorbance of the solution was measured once every 0.5 h. The absorbance of the RhB solution was examined by a UV–vis spectrophotometer (UV-3150, Shanghai, China). The absorbance peak of RhB was recorded at 554 nm. The decrease absorbance of RhB is used as a measure of the photocatalytic degradation. The degradation was calculated according to the Equation (1):

(1) $$\mathrm{Degradation}\;\mathrm{Efficiency}\;(\%)=(1\;-\frac{C}{C_{0}})\;\times \;100\%=(1\;-\frac{A}{A_{0}})\;\times \;100\%$$

where C_0_ is the concentration before degradation and C is the concentration after the degradation of RhB, respectively. A_0_ and A are, respectively, known as the initial and instant RhB absorbance.

## 3. Results and Discussion

**Figure 1:**
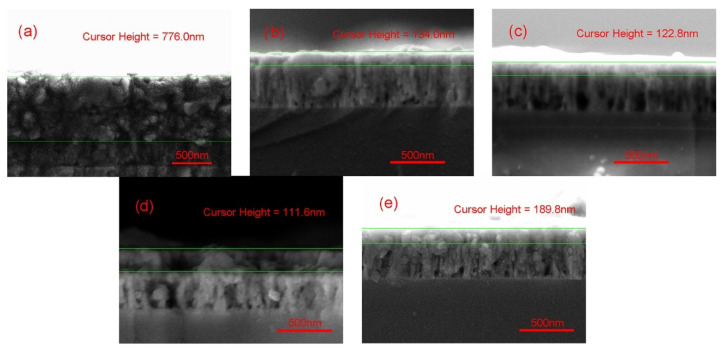
SEM side-view images of ZnO/TiO_2_ films: (**a**) ZT1; (**b**) ZT2; (**c**) ZT3; (**d**) ZT4; (**e**) ZT5.

The thickness of as-prepared TiO_2_/ZnO nano-films was detected by SEM side-view (Figure 1a–e). The underlying ZnO films had a similar thickness of approximately 373 nm. The thickness of outer TiO_2_ in ZT1, ZT2, ZT3, ZT4, and ZT5 were about 776.0 nm, 134.0 nm, 122.8 nm, 111.6 nm, and 189.8 nm, respectively. Further, the average deposition rates of the mentioned samples were calculated to be 12.93 nm/min, 2.23 nm/min, 2.05 nm/min, 1.86 nm/min, and 3.16 nm/min, respectively. The average deposition rate decreased with the increasing O_2_/Ar ratio, which was due to the occurrence of transition from metallic to reactive sputtering modes. The TiO_2_ and ZnO thin films exhibit effective contact and uniform thickness, suggesting that TiO_2_/ZnO heterojunction was produced during the layer-by-layer sputtering process.

**Figure 2:**
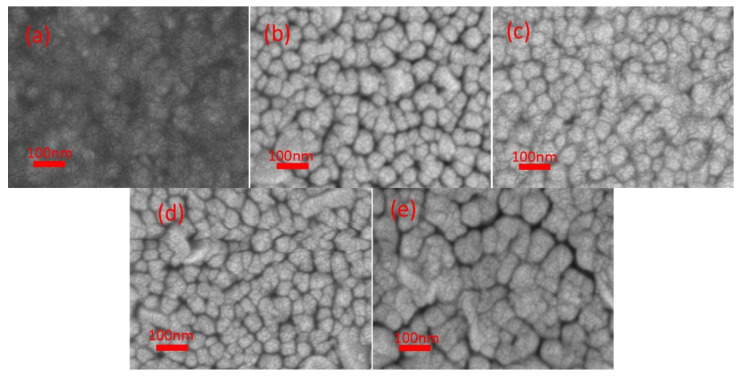
SEM top-view images of ZnO/TiO_2_ films: (**a**) ZT1; (**b**) ZT2; (**c**) ZT3; (**d**) ZT4; (**e**) ZT5.

The morphologies of as-prepared samples were characterized by SEM top-view (Figure 2). The nano-sized spherical particles have appeared on all the films. The ZT1 was composed of small and dense spherical particles, which may ascribe to its large thickness and the considerable formation of rutile TiO_2_. Moreover, it is also shown that the outer TiO_2_ particles would grow bigger with the increase of O_2_/Ar ratio from 5:35 to 20:35. In addition, asymmetrical clusters with the elongated shape were observed in ZT4, which were hard to find in ZT2 and ZT3. The deposition rate is taken into account to explain the variation of TiO_2_ particles. Due to the lower deposition rate caused by the increased O_2_/Ar ratio, the particles have enough time to fuse together at the inter-particle contact, resulting in the growth of particles and the formation of asymmetrical clusters. It is observed that the ZT5 obtained by using a ceramic TiO_2_ target showed a cracked surface with spherical particles of varying sizes. Different sizes and distributions of nanospheres on the sample surface suggest that the target type and O_2_/Ar ratio may be crucial for film microstructure and morphology.

**Figure 3:**
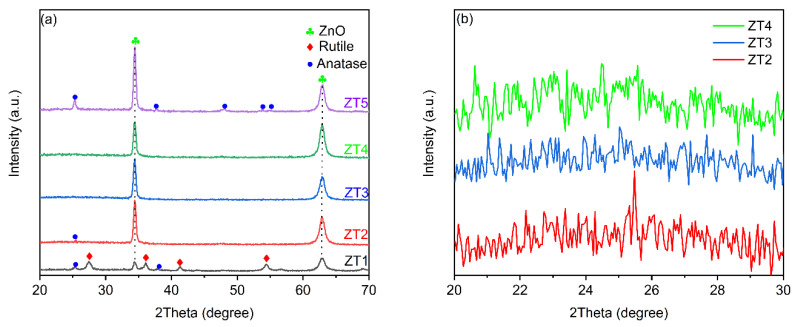
(**a**) XRD patterns of ZT1, ZT2, ZT3, ZT4, and ZT5; (**b**) Zoomed-in details of ZT2, ZT3 and ZT4.

The crystalline structure of TiO_2_/ZnO nano-films was investigated by XRD patterns (Figure 3a). There were two peaks at 2θ = 34.4° and 62.9° in all the films, corresponding to (002) and (103) planes of ZnO, respectively. Thus, ZnO nanostructures have a wurtzite crystal structure. Moreover, the intensity ratio values of I_(0 0 2)_/I_(1 0 3)_ varied with the rising values of O_2_/Ar. This result indicates that the orientation growth of crystalline ZnO could be changed by the variation of O_2_/Ar flow ratio during the process of depositing TiO_2_. Moreover, the intensities of 34.4° and 62.9° for ZT1 were much weaker than the others because the outer TiO_2_ layer was thicker and the signal strength of the underlying ZnO layer was weakened. As for the ZT1, XRD peaks of the rutile TiO_2_ located at 27.4°, 36.1°, 54.4°, and 28.1° can be assigned to the (100), (101), (221), and (220) plane. The two weak peaks located at 25.4° and 37.7° belonged to the (110) and (004) of anatase TiO_2_. The proportion of rutile was calculated by the Equation (2) [26]:

(2) $$\mathrm{Rutile}\;\mathrm{Content}=\frac{1}{1+0.8\frac{I_{A}}{I_{R}}}$$

where I_A_ and I_R_ represent the X-ray intensities of the anatase and rutile peaks at 25.4° and 27.4°, respectively. The proportion of rutile was 72.5%, suggesting that a substantial fraction of rutile TiO_2_ nanoparticles was fabricated in the outer layer, which was well consistent with SEM results. With the increase of O_2_/Ar flow ratio, there was only a weak peak of the anatase TiO_2_ located at 25.4° for ZT2, which indicates that the crystallinity of TiO_2_ in ZT2 is weak. Furthermore, no peak characteristic of crystalline TiO_2_ could be observed for ZT3 and ZT4 (Figure 3b), which may be due to the extremely thin thickness of the outer TiO_2_ layer in ZT3 and ZT4. For ZT5, XRD peaks located at 25.4°, 37.7°, 47.9°, 53.9°, and 55.1°, respectively, corresponding to the peak anatase TiO_2_ (110), (004), (200), (105), and (211) and there were no presence of peaks corresponding to the anatase. The annealing temperature for the anatase phase to change into the rutile phase is about 600 to 800 °C [27]. Furthermore, TiO_2_ is liable to crystallize and transform to rutile with the increase of the thickness [28,29]. However, it is worth noting that ZT5 has similar thickness with ZT2, ZT3, and ZT4. Thus, the chemical composition of TiO_2_ before annealing also affects the crystallization progress.

**Figure 4:**
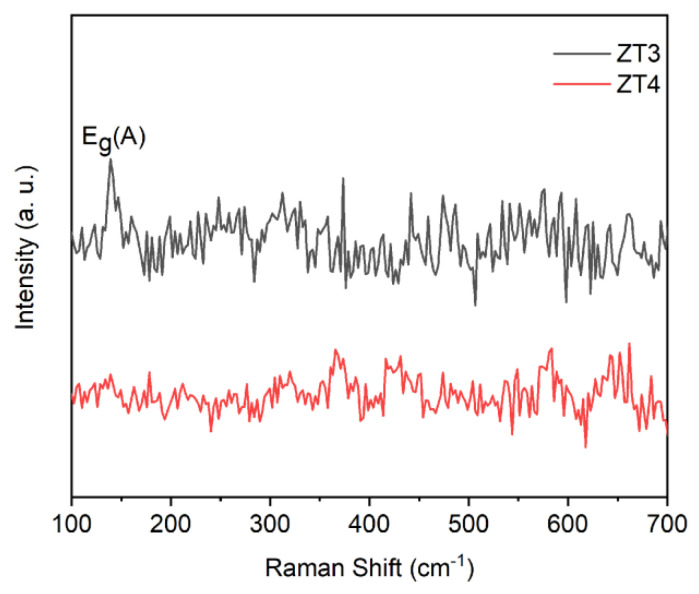
Raman spectrum of ZT3 and ZT4.

Raman was used for further exploring the crystal structure of ZT3 and ZT4. The Raman spectra of ZT3 and ZT4 were given in Figure 4. Figure 4 shows that there is no distinct peak corresponding to any of the crystalline phases of TiO_2_ in ZT4 and only a weak peak at 144 cm^−1^ responding to the Eg(A) mode of anatase TiO_2_ [30], indicating that the ZT4 consists of ZnO and amorphous TiO_2_ and the upper TiO_2_ layer in the ZT3 is composed of amorphous TiO_2_ and a small fraction of anatase. Thus, the XRD pattern of ZT3 shows no peak characteristic of crystalline TiO_2_ because the film is really thin and does not ascribe to an amorphous TiO_2_ layer. A similar phenomenon was found by other groups [31].

**Figure 5:**
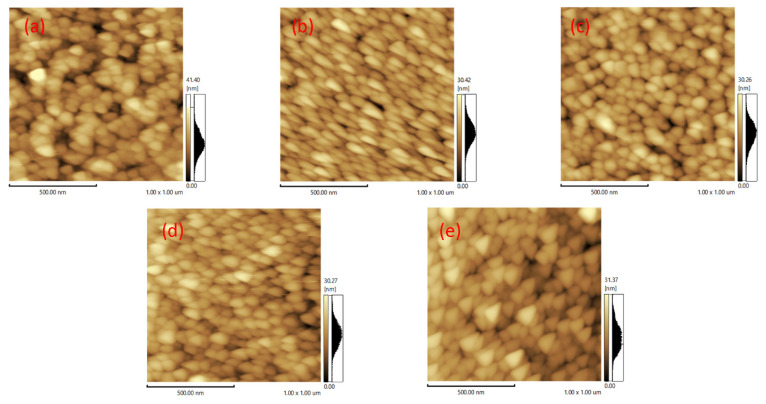
AFM side-view images of ZnO/TiO_2_ films: (**a**) ZT1; (**b**) ZT2; (**c**) ZT3; (**d**) ZT4; (**e**) ZT5.

AFM was used to investigate the surface properties of the as-prepared films. Figure 5 shows the 2D AFM images of the thin films. The average surface roughness (Ra) values of ZT1, ZT2, ZT3, ZT4, and ZT5 were 5.01 nm, 3.09 nm, 3.27 nm, 2.86 nm, and 2.84 nm. The ZT5 had the largest Ra, which was regarded as the result of the heavy thickness of TiO_2_. Apart from ZT1, the Ra value of the ZT3 was larger than that of other samples. Relatively large surface roughness increases the contact area between the TiO_2_ nanospheres and contaminants, thus providing a larger specific surface area and more active sites for photocatalytic reactions. The largest Ra of the ZT5 is attributed to the large thickness and the rutile in the ZT5 can act as recombination sites. This fact indicates that the ZT3 with the Ra value of 3.27 nm may show more outstanding photocatalytic performance than other samples in this work.

**Figure 6:**
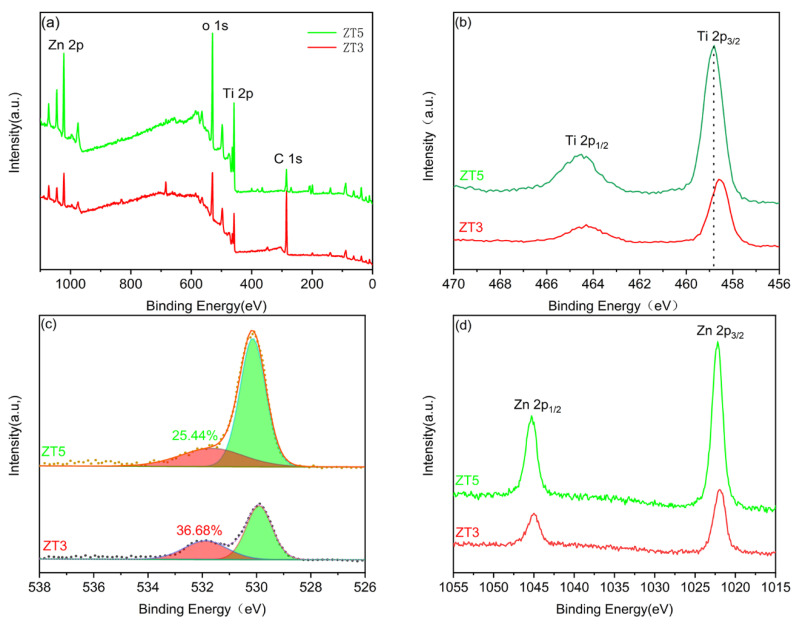
(**a**) Survey spectra; (**b**) Ti 2p; (**c**) O1s; and (**d**) Zn 3d XPS spectra of as-prepared samples.

The XPS spectra of ZT3 were shown in Figure 6a–d, coupled with ZT5 for comparison. The peaks of Ti 2p, O 1s, and Zn2p were clearly observed in Figure 6a. Furthermore, the C1s peak at 284.8 eV was attributed to adventitious carbon species, which were inevitable during the production and testing process. The high-resolution Ti 2p spectra of ZT3 and ZT5 were displayed in Figure 6b. The ZT5 showed two peaks located at 458.8 and 464.5 eV, which were corresponded to Ti 2p_1/2_ and Ti 2p_3/2_, respectively. The separation gap between Ti 2p_1/2_ and Ti 2p_3/2_ was 5.7 eV, demonstrating the existence of Ti^4+^ valence state in ZT5. In comparison, a small negative shift of the Ti 2p peaks about 0.2 eV can be observed for ZT3, which could ascribe to the change in the surrounding bonding environment caused by the formation of Ti^3+^ [32]. Hence, the presence of Ti^3+^ for ZT3 fabricated at the O2/Ar ratio of 10:35 is assuredly confirmed [33]. The O 1s peak for ZT5 can be divided into two peaks. The main peak was located at 529–530 eV, which was assigned to the lattice oxygen in the ZnO/TiO_2_ nano-film. A shoulder peak at 531–532 eV was observed, resulting from OH species and adsorbed molecular oxygen. Thus, the generation of surface OVs for the ZT3 during the sputtering and annealing process is confirmed. Furthermore, the content of surface OVs can be indirectly measured by the relative area of the corresponding peaks. As clearly shown in Figure 6c, the relative concentration of ZT3 (36.68%) is higher than that for ZT5 (25.44%). Thus, the generation of surface OVs for the ZT3 during the sputtering and annealing process is confirmed. Meanwhile, the results of the high-resolution Zn 2p XPS spectrum for both ZT3 and ZT5 were showed in Figure 6d. For ZT5, the Zn 2p_3/2_ and 2p_1/2_ peaks centered at 1022.0 and 1045.1 eV, which corroborated the Zn^2+^ valence state. It is to be noted that the peaks of Zn 3d in ZT3 are similar to those in ZT5, indicating that the chemical state of Zn in the as-prepared samples is invariable. The composition of ZT3 and ZT5 are shown in Table 1.

**Figure 7:**
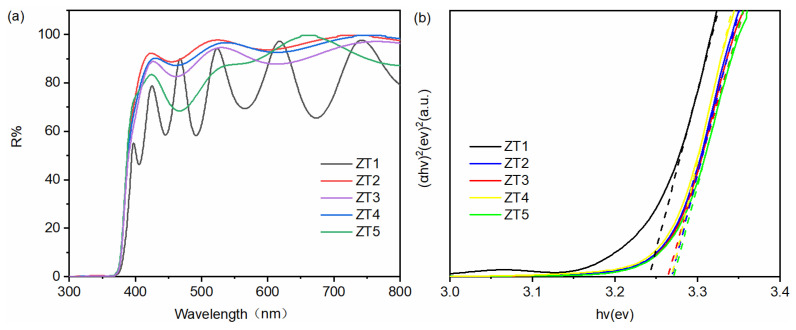
The transmittance (**a**) and the bandgap values (**b**) of the ZT1, ZT2, ZT3, ZT4, and ZT5.

Figure 7a shows the transmission spectra of the TiO_2_/ZnO nano-films. It is worth noting that the average transmittance of ZT1 is smaller than that of the other samples, which could be ascribed to the heavy thickness of ZT1, resulting in the increasing optical scattering. The bandgap values (E_g_) are obtained using the Equation (3):

(3) $${\left(\alpha h\nu \right)}^{2}=A\left(h\nu -E_{g}\right)$$

where α is the absorption coefficient, h is the Planck constant, ν is the light frequency, and A is a proportionality, respectively. As shown in Figure 7b, the bandgap values are 3.25 eV, 3.27 eV, 3.26 eV, 3.26 eV, and 3.27 eV for ZT1, ZT2, ZT3, ZT4, and ZT5. Furthermore, it is to be noted that the forbidden bandwidth of the ZT1 (3.25 eV) is slightly narrower than others. The production of the rutile TiO_2_ should be responsible for this phenomenon and the excessive fraction of rutile helps to accelerate the rate of e–h recombination, leading to low photocatalytic degradation. There is no significant change in the band gap, indicating that the absorption ranges of the samples are not the main role that affects the photocatalytic performance in this work.

**Figure 8:**
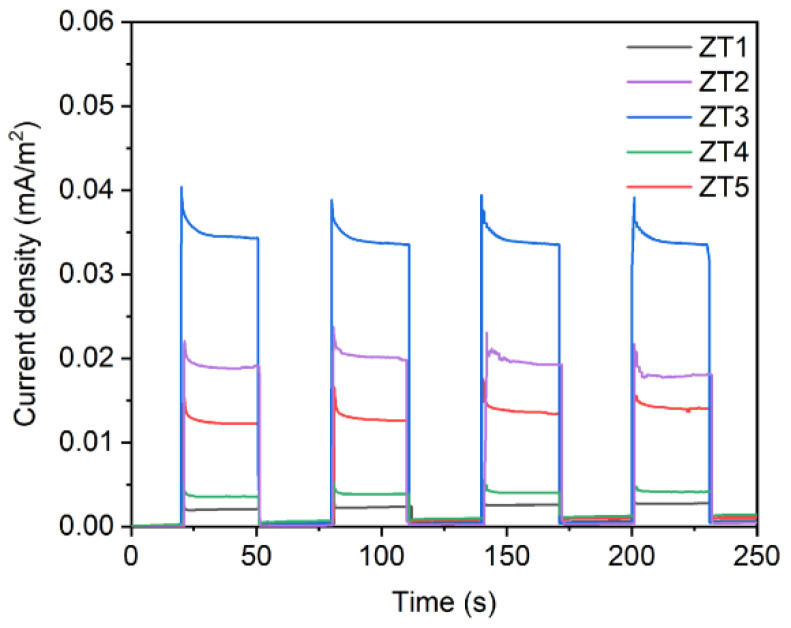
Transient photocurrent responses of the as-prepared simples.

Figure 8 shows the photocurrent response of the prepared photocatalytic films in the intermittently switched lamp state. Carriers generate in photocatalysts in presence of light accompany with current. A larger photocurrent means a slower recombination of photo-generated charge carriers and a faster interfacial change–transfer process, which will ultimately optimize the photocatalytic properties of the films. The photocurrent densities of the ZT1, ZT2, ZT3, ZT4, and ZT5 are 0.002 mA/cm^2^, 0.019 mA/cm^2^, 0.034 mA/cm^2^, 0.004 mA/cm^2^ and 0.013 mA/cm^2^, respectively. All the TiO_2_/ZnO nano-films are very sensitive to the light, indicating that the quality of the samples was high. Moreover, it was impressive that the photocurrent intensity of ZT3 was dramatically higher compared with that of others. The photocurrent density of the ZT3 was 17.0 times and 2.6 times higher than that of ZT1 and ZT5, respectively. It could suggest that the suitable surface roughness and the existence of Ti^3+^/OVs can improve the separation and transfer efficiency of photo-induced e–h pairs. Meanwhile, the smallest photocurrent intensity of ZT1 is attributed to the large thickness of the outer TiO_2_ layer in ZT1 and the production of a high fraction of rutile TiO_2_, which provide many recombination sites of photo-generated e–h pairs and reduce the separation efficiency of charge carriers.

**Figure 9:**
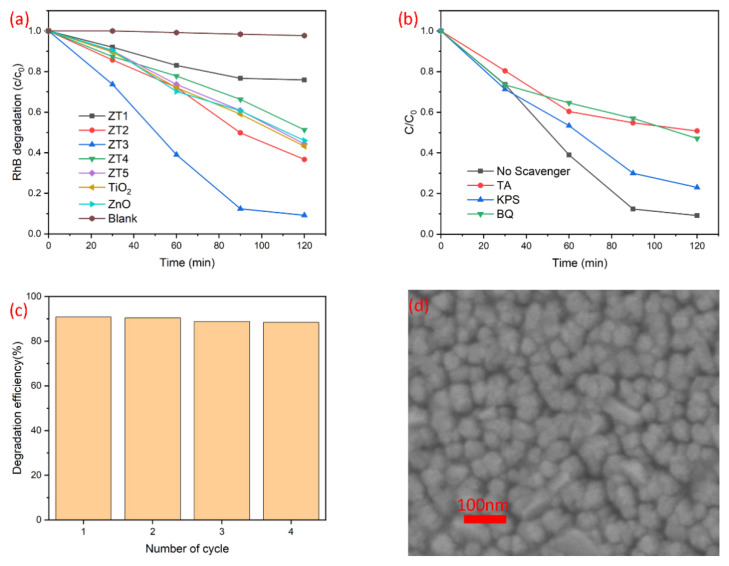
(**a**) The photocatalytic degeneration of RhB over ZT1, ZT2, ZT3, ZT4, and ZT5; (**b**) the photocatalytic degeneration of RhB with various scavengers over ZT3; (**c**) the stability of the ZT3; and (**d**) the SEM image of ZT3 after the photocatalytic test.

The photocatalytic ability of the TiO_2_/ZnO nano-films was monitored for the degradation Of RhB under UV-light illumination (Figure 9a). Bare ZnO nano-film and TiO_2_ nano-film were used as control samples. The preparation process was the same as ZT3 but in absence of the TiO_2_ layer and the ZnO layer, respectively. It was found that the ZT3 exhibited remarkably superior photocatalytic activity, by removing 88.0% RhB in 90 min duration and 90.8% RhB in 120 min duration. Hence, the ZnO coupled with the TiO_2_ through magnetron sputtering by using a metallic Ti target at an O_2_/Ar ratio of 10:35 could dramatically enhance the photocatalytic performance of TiO_2_ heterojunction under UV light illumination. Hole scavenger (tartaric acid, TA), electron scavenger (potassium persulphate, KPS), and superoxide anion radical scavenger (p-Benzoquinone, BQ) were added to ascertain the photocatalytic mechanism. As shown in Figure 9b, hole and superoxide anion radical play the dominant role. The reusability of the nano-films was tested, ZT3 removed 88.4% RhB in 120 min after the fourth ring (Figure 9c). The surface of nano-films did not change significantly after the photocatalytic test (Figure 9d).

**Figure 10:**
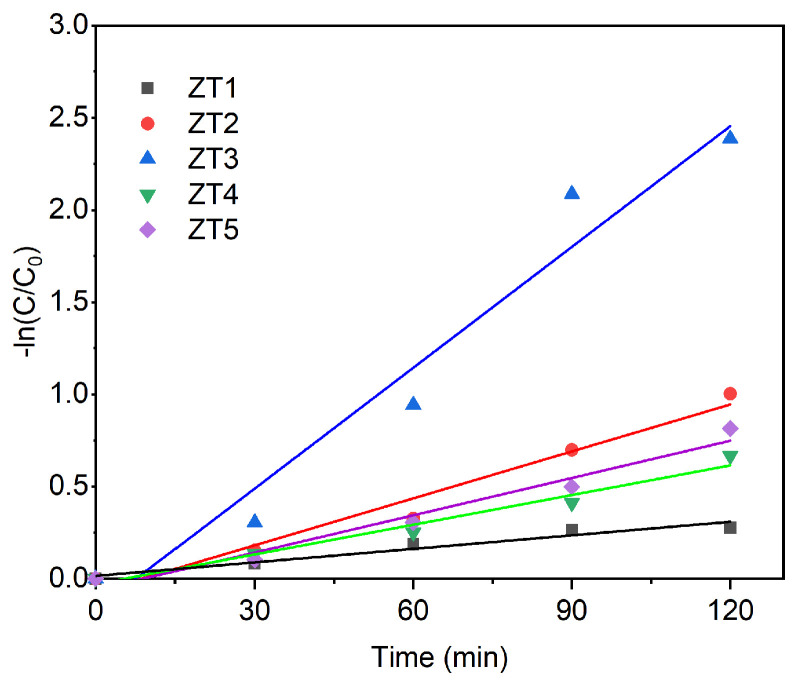
The reaction rate constants of ZT1, ZT2, ZT3, ZT4, and ZT5. The reaction rate constants of ZT1, ZT2, ZT3, ZT4, and ZT5.

Figure 10 shows the pseudo-first-order kinetic curves of the reaction rate. The reaction rate constant (k_app_) could be described with the Equation (4):

(4) $$-\ln\left(\frac{C}{C_{0}}\right){=k}_{\mathrm{app}}\times \;t$$

where the C is the RhB concentration at irradiation time (t) and C_0_ is the initial RhB concentration. The k_app_ values of ZT1, ZT2, ZT3, ZT4, and ZT5 were 2.5 × 10^−3^ min^−1^, 8.5 × 10^−3^ min^−1^, 21.9 × 10^−3^ min^−1^, 5.4 × 10^−3^ min^−1^, and 6.7 × 10^−3^ min^−1^, respectively. It is worth noting that the reaction rate constant of ZT1 was 8.76 times and 3.22 times higher than that of ZT1 and ZT5, respectively, indicating that depositing the TiO_2_ through magnetron sputtering using a metallic Ti target at O_2_/Ar ratios of 10:35 is a better strategy to couple with the ZnO compared with using a metallic Ti or ceramic TiO_2_ in Ar gas flow. Moreover, photocatalytic degradation ability of dye for ZT3 was compared with other recent similar reports [22,23,34,35,36,37,38], which are shown in Table 2. Accordingly, the ZT3 is a superior photocatalytic film for wastewater treatment.

**Figure 11:**
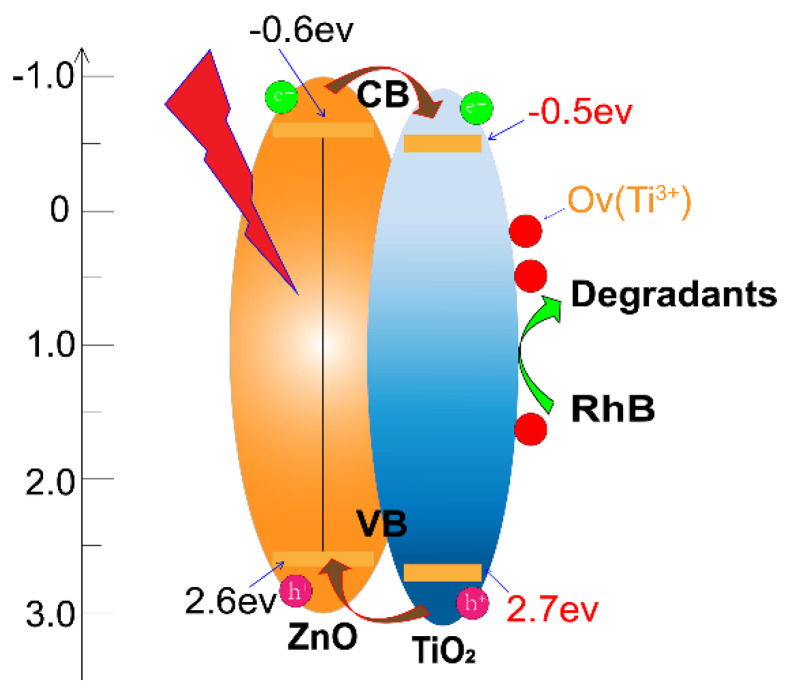
ZnO/TiO_2_ film degradation RhB solution mechanism.

Based on the results above, the improvement in photoreaction of the ZT3 may be caused by multiple contributions. In Figure 11, the conduction band (E_CB_ = −0.5 eV) and valence band (E_VB_ = 2.7 eV) of TiO_2_ [39] are marginally higher than those of ZnO (E_CB_ = −0.4 eV and E_VB_ = 2.6 eV) [40] so that the electrons generated by ZnO tended to transfer to the conduction band of TiO_2_ through the interface. Meanwhile, the recombination of photo-generated e–h pairs on the surface was suppressed by the production of OVs, contributing to more efficient charge transportation and improved photocatalytic efficiency compared with the other samples. Moreover, the appropriate thickness and well-defined crystalline structure of TiO_2_ in the upper layer of ZT3 play a significant role, facilitating the transport of photo-generated e–h pairs from bulk to the surface. Furthermore, the uniform morphologies and suitable Ra of ZT3 may provide a large interfacial contact area with contaminants and prolong the lifetime of charge carriers, which is helpful for the photocatalytic process.

## 4. Conclusions

The ZT3 with outstanding photocatalytic performances was synthesized by a simple magnetron sputtering method. The XPS results confirmed that the formation of surficial OVs for ZT3. The ZT3 removed 88.0% rhodamine B in 90 min duration and 90.8% RhB in 120 min duration. The reaction rate constant value of ZT3 was 21.9 × 10^−3^ min^−1^, which was 8.76 times and 3.22 times higher than that of ZT1 and ZT5. The improved photocatalytic properties were achieved by combining the heterojunction interface and surface OVs. Besides, suitable thickness, well-ordered crystal structure, and proper roughness of ZT3 also played an important role in the photocatalytic process. The strategy that combines with constructing heterojunction and inducing surface OVs might provide a new approach to design photocatalytic films for the treatment of contaminated water.

## Figures and Tables

**Table 1:** The composition of ZT3 and ZT5.

Samples	Ti (at%)	Zn (at%)	O (at%)
ZT3	24.52	5.59	68.89
ZT5	22.87	4.63	72.49

**Table 2:** Comprehensive comparison of the ZT3 with other recent similar photocatalytic films.

Samples	Dye Concentration	Removal	k_app_ (10^−3^ min^−1^)
(This work)	RhB (5 mg/L) in 50 mL	90.8% in 2 h	21.9
CNT-ZnO Composition [34]	Methyl orange	41.5% in 4 h	2.6
Ag-loaded TiO_2_-ZnO film [35]	Methyl blue	80% in 2 h	12.6
Ag-Ag_2_O-TiO_2_ film [23]	RhB (5 mg/L) in 50 mL	68.7% in 6 h	-
Ag/TiO_2_ film [24]	RhB (5 mg/L)	72.1% in 6 h	3.2
TiO_2_-ZnO film [36]	Methyl blue (10 ppm)	44% in 2 h	-
Bi_2_O_3_/TiO_2_ film [37]	Methyl blue (10–5 M)	40% in 6 h	1.32
Cr doped TiO_2_ film [38]	Methyl blue (3 mg/L)	75% in 2 h	11.3

## Data Availability

The data are not publicly available due to legal or ethical reasons.
